# Identification and Targeting of Mutant Neoantigens in Multiple Myeloma Treatment

**DOI:** 10.3390/curroncol30050348

**Published:** 2023-04-29

**Authors:** Valentina Urzì Brancati, Letteria Minutoli, Herbert Ryan Marini, Domenico Puzzolo, Alessandro Allegra

**Affiliations:** 1 Department of Clinical and Experimental Medicine, University of Messina, 98125 Messina, Italy; valeurzi@hotmail.it (V.U.B.); hrmarini@unime.it (H.R.M.); 2 Department of Biomedical and Dental Sciences and Morphofunctional Imaging, University of Messina, 98125 Messina, Italy; puzzolo@unime.it; 3 Division of Haematology, Department of Human Pathology in Adulthood and Childhood, University of Messina, 98125 Messina, Italy; aallegra@unime.it

**Keywords:** multiple myeloma, neoantigens, conjugated-drug antibodies, bispecific antibodies, trispecific antibodies, CAR-T cells

## Abstract

Multiple myeloma (MM) is malignant disease characterized by the clonal proliferation of plasma cells in the bone marrow, leading to anemia, immunosuppression, and other symptoms, that is generally hard to treat. In MM, the immune system is likely exposed to neoplasia-associated neoantigens for several years before the tumor onset. Different types of neoantigens have been identified. Public or shared neoantigens derive from tumor-specific modifications often reported in several patients or across diverse tumors. They are intriguing therapeutic targets because they are frequently observed, and they have an oncogenic effect. Only a small number of public neoantigens have been recognized. Most of the neoantigens that have been identified are patient-specific or “private”, necessitating a personalized approach for adaptive cell treatment. It was demonstrated that the targeting of a single greatly immunogenic neoantigen may be appropriate for tumor control. The purpose of this review was to analyze the neoantigens present in patients with MM, and to evaluate the possibility of using their presence as a prognostic factor or as a therapeutic target. We reviewed the most recent literature on neoantigen treatment strategies and on the use of bispecific, trispecific, and conjugated antibodies for the treatment of MM. Finally, a section was dedicated to the use of CAR-T in relapsed and refractory patients.

## 1. Introduction

Multiple myeloma (MM) is a hematologic disorder characterized by the accumulation of clonal plasma cells (PCs) in the bone marrow (BM). This causes an abnormal production of monoclonal immunoglobulin (M-protein), easily detectable in urine and/or blood. Moreover, the presence of clonal cells suppresses the normal plasma cell population, causing immunosuppression, abnormal hematopoiesis, lytic bone lesions, and, finally, impaired renal function [1]. MM represents 1% of all cancers, and it is the second most frequent hematologic malignancy, with an incidence estimated in 4.5–6/100.000/year [2]. At diagnosis, patients have a median age of 66–70 years, with a slightly higher prevalence in men. MM is generally not considered a genetic disease, but few familial cases have been observed and close relatives have a six times higher risk of developing MM than non-related adults [3].

Usually, MM progresses from an asymptomatic stage named monoclonal gammopathy of undetermined significance (MGUS). It has been estimated that 1% per year of patients with MGUS evolve toward MM. Some patients show an intermediate pre-malignant stage, also asymptomatic but more advanced, known as smoldering (or indolent) multiple myeloma (SMM). Amongst SMM patients, it has been estimated that 10% progress to MM per year during the first 5 years after diagnosis, 3% during the successive 3 years, and 1.5% per year after that [4].

To make a diagnosis of MM, the following must be evaluated: (i) the monoclonal component with serum and/or urine protein electrophoresis; (ii) BM infiltration of plasma cells; (iii) possible lytic bone lesions; (iv) blood cell counts, with differential creatinine and calcium levels in blood serum [5].

Basing on these criteria, serum M-protein levels (<10%) are used for MGUS diagnosis, and there are no end-organ damage or myeloma-defining events (MDEs) [6]. In SMM, higher levels of M-protein and/or presence of clonal plasma cells in the BM are evident, while, similarly to MGUS, there are no end-organ damage and MDEs [7]. To diagnose symptomatic MM, instead, there must be the presence of ≥30% BM clonal plasma cells or a biopsy-proven bony or extramedullary plasmacytoma, and at least one MDE, such as end-organ damage, or any biomarkers of malignancy (60% clonal BM plasma cells, involved/uninvolved ratio of serum free light chain ≥100, at least one focal lesion ≥5 mm on magnetic resonance images) [5].

Until the early 2000s, therapy options for MM were limited, and the patients’ survival rate at five years was <35.6% [8]. During the past 20 years, eligible patients have been treated with high-dose therapy followed by autologous hematopoietic cell transplantation (auto-HCT). Furthermore, the use in frontline therapy of immunomodulatory drugs (IMiDs), such as lenalidomide, proteasomal inhibitors (PIs), e.g., bortezomib, and/or monoclonal antibodies, has obtained better clinical outcomes, overall survival (OS), and progression-free survival (PFS) in both newly diagnosed (NDMM) or relapsed/refractory (RRMM) subjects [9,10,11]. However, despite the improvements in therapy and care, MM remains hard to treat, with only a minority of patients achieving long-term disease control, while most of the patients are likely to relapse and often show resistance to previously used agents [12,13]. This highlights the need to broaden the therapeutic options for MM patients, exploring novel mechanisms of actions. In this context, tumor-associated neoantigens, to which the immune system is likely exposed for many years before MM onset, bispecific, trispecific, and conjugated antibodies and chimeric antigen receptor (CAR) T cells are promising therapeutic strategies. In this review, we analyzed the neoantigens showed by MM patients, describing the possibility of using their presence as a prognostic factor or as a therapeutic target. We also reviewed the most recent studies on the use of bispecific, trispecific, and conjugated antibodies, and we dedicated a section to the use of CAR-T in RRMM patients.

## 2. Neoantigens and Neoantigen Vaccination Strategies

Neoantigens are peptides abnormally expressed in cancer cells. They originate from the genetic instability always taking place during carcinogenesis. T cell receptors (TCRs) can recognize neoantigens after their presentation on the cell surface, thus, eliciting a T cell-mediated anti-tumor immune response. There are two main categories of tumor antigens: tumor-associated antigens (TAAs) and tumor-specific antigens (TSAs) [14]. TAAs are proteins produced by non-mutated genes that are significantly over-augmented in cancer cells, but rarely present in normal cells. As TAAs are “self” peptides, they are subject to both central and peripheral tolerance mechanisms [15], and their targeting may cause autoimmune toxicity. Differently, TSAs are neoantigens deriving from somatic mutations, present in tumor cells, but not in normal ones. Therefore, the immune system considers them as non-self, and neoantigen-specific immune responses are not affected by tolerance. Furthermore, targeting TSAs does not frequently lead to autoimmunity reactions [16]. This explains why neoantigens are considered ideal targets for therapeutic cancer vaccines and T cell-based cancer immunotherapy. Neoantigens can be classified into public or shared neoantigens and private or personalized neoantigens. Public neoantigens originate from tumor-specific mutations, or driver mutations, and are frequently found across cancer patients in different malignancies [17]. They are considered attractive therapeutic targets, but only a few of them have been found. On the contrary, private neoantigens, which represent the largest part of identified neoantigens, derive from non-recurrent driver mutations, or passenger mutations, and can be considered patient-specific [18].

**Figure 1:**
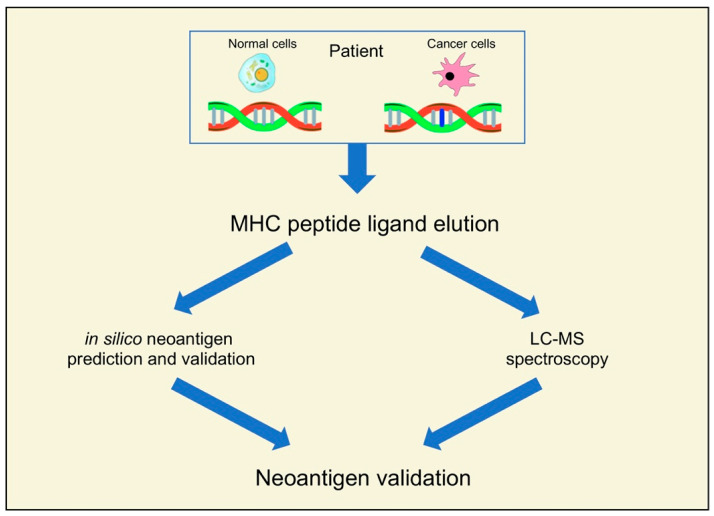
Recent pipelines for the identification of neoantigens.

The identification of neoantigens with high throughput has been made possible by new advances in next-generation sequencing (NGS) and bioinformatic tools and pipelines [19,20,21]. Neoantigens in NGS data are primarily predicted using in-silico methods and LC-MS spectroscopy (Figure 1).

These methods start by finding tumor-specific genetic changes in protein-coding areas. This is generally achieved by using deep coverage short-read WES of matched tumor and germline DNA [22,23]. DNA-seq methods can be used to identify in an accurate way neoantigens resultant from missense and frame-shift mutations, indels, translocations, gene fusions, and other structural alterations [24,25]. Likewise, neoantigens from RNA modifications, such as RNA editing events and alternative splicing, can be found with mRNA or whole transcriptome RNA-seq techniques [26,27]. While potential peptides are only found in particular HLA alleles [28,29,30], accurate neoantigen prediction is only possible after the identification of tumor-specific genetic changes. For a precise identification of immunogenic candidate neoantigens, functional validation of predicted neoantigens is essential, particularly in the case of putative neoantigens generated by in silico pipelines [31].

Concerning neoantigens and MM, several oncogenic driver mutations have been documented, affecting signaling pathways mediated by *MEK/ERK*, *KRAS*, *NRAS*, *BRAF*, and *NF-κB*, and also affecting genes involved in epigenetic regulation, such as *HIST1H1E*, *KMT2C*, and *CREBBP* [1,32]. The relationship between neoantigen load, MM prognosis, and response to therapy has been the object of some recent studies (Table 1). Miller et al. studied the relationship between neoantigen burden and therapy-response, observing that patients with a higher neoantigen load had a significantly shorter PFS compared with patients with a low neoantigen burden [33]. Dong et al. [34] confirmed these results, reporting that aberrant intron retention in MM patients caused high levels of neoantigens, with a worse overall survival (OS). Moreover, a study by Perumal et al. [22] showed that relapsed MM patients had a higher neoantigen burden. They also recognized shared neoantigens among many patients in three driver oncogenic genes in MM (*KRAS*, *NRAS*, and *IRF4*), indicating the likely presence of shared neoantigens in MM. Jian et al. [35] characterized the quality of immune response to neoantigens, and for this reason they developed an immune response score (NAIRscore), taking in consideration the neoantigen load, the cytolytic score, and the HLA-I score. They studied 478 patients and found that a high NAIRscore was associated with increased OR, reflecting the underlying lower driver-gene mutations and downregulated immune response.

The adaptive immune system can identify peptides that correlate to somatic changes in tumor cells as “non-self” neoantigens [36]. Due to the ability of mature T cells to identify tumor-specific mutations as neo-antigens, they are attractive targets for cancer immunotherapy. More mutated tumors are more likely to produce neoepitopes, which tumor-infiltrating T cells may identify. As a result, immune therapies are more effective against malignancies with high mutation rates [22]. Neoantigen-specific T cell response expansion may act as direct pharmacodynamic biomarkers of immunotherapeutic interventions in MM, and shared neoantigens may be the focus of therapeutic strategies. The potential to use neoantigens as targets for a variety of therapeutic approaches, including conjugated, bispecific, and trispecific antibodies, as well as CAR-T, will be covered in the following sections.

## 3. Conjugated, Bispecific and Trispecific Antibodies

**Figure 2:**
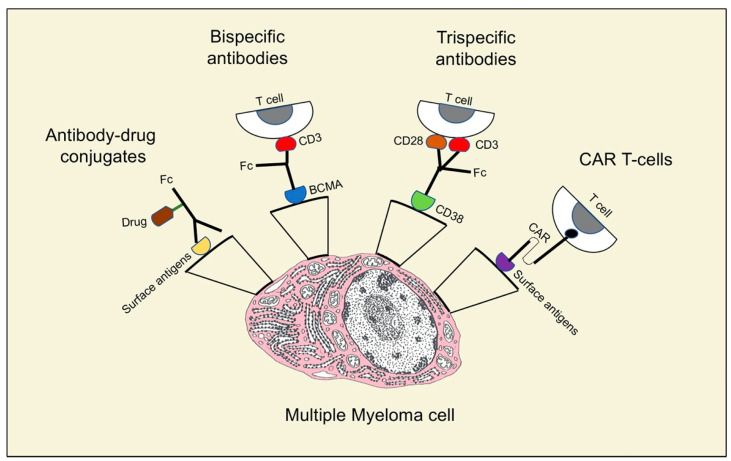
Different structures and mechanisms of action of neoantigens in multiple myeloma treatment.

As indicated above, antibody–drug conjugates and bispecific and trispecific antibodies are included in the relevant therapeutic strategies for MM patients (Figure 2).

### 3.1. Antibody-Drug Conjugates

Antibody–drug conjugates (ADCs) act by adding a potent cytotoxic agent to the monoclonal antibody via a stable linker (Figure 2). The amount of toxic agent is defined by the drug/antibody ratio (DAR). After the bond with a cell surface antigen, the antibody enters the cell by receptor-mediated endocytosis and is then transported to the lysosomes where the linker is separated, facilitating the intracellular release of the cytotoxic substance. Given this mechanism of action, the toxic agent targets antigen-expressing cells, while sparing other cells and reducing systemic toxicities [37]. In August 2020, the Food and Drug Administration approved belantamab mafodotin for RRMM patients, an ADC anti- B cell maturation antigen (BCMA) linked to the inhibition of microtubule monomethyl auristatin [38]. BCMA, a transmembrane protein belonging to the tumor necrosis factor (TNF) superfamily, regulates B cell maturation and their differentiation into plasma cells [39]. It is an ideal target for antibody-mediated treatments, because it is expressed primarily on plasma cells, yet is essentially absent on naive and memory B cells. Trudel et al. [40] evaluated tolerability, safety, and preliminary clinical activity of belantamab mafodotin GSK2857916 in a population of 73 patients (38 patients in the dose-escalation part 1 and 35 patients in the dose-expansion part 2). No maximum tolerated dose (MTD) was identified in part 1. Common adverse events (AEs) were corneal lesions, thrombocytopenia, and anemia. Twelve serious treatment-related AEs and no treatment-related deaths were registered, and the OR was 60%. After an additional 14 months of follow-up [41], the same authors observed a 60% OR on the 35 patients in the dose-expansion part 2. The median PFS and median duration of response were 12 months and 14.3 months, respectively. They still reported thrombocytopenia and corneal events and did not identify new safety signals. Other ADCs are in clinical trials for RRMM, with some of them bound to BCMA as well, such as AMG224, Medi2228, and CC99712. Other ADCs target CD38 (TAK-573 and TAK-169), CD138 (indatuximab ravtansine, BT062), and CD56 (lorvotuzumab mertansine). Indatuximab is an anti-CD138 antibody linked to the microtubule inhibitor DM4; it was assessed in two studies.

In the first study [42], patients were treated with indatuximab ravtansine intravenously and lenalidomide and dexamethasone in phase 1, while they were given the recommended phase 2 dose of indatuximab ravtansine in combination with lenalidomide and dexamethasone in phase 2. The protocol was modified to include treatment with indatuximab ravtansine plus pomalidomide and dexamethasone in additional patients more heavily pre-treated than the indatuximab ravtansine plus lenalidomide group. Phase 1 had the main endpoint of establishing indatuximab ravtansine dose-limiting toxicities and MTD, and to define the objective response rate (ORR) and clinical benefit response for phase 2. The MTD of indatuximab ravtansine plus lenalidomide reported for the 64 participants was 100 mg/m^2^. The indatuximab ravtansine plus lenalidomide group and the indatuximab ravtansine plus pomalidomide group had an ORR of 71.7% and 70.6%, respectively. A clinical benefit response was shown in 85% of the indatuximab ravtansine plus lenalidomide group and in 88% of the indatuximab ravtansine plus pomalidomide group. Moreover, both groups showed neutropenia, anemia, and thrombocytopenia as the most common AEs. Lastly, 35 patients showed treatment-emergent adverse events (TEAEs) that led to therapy suspension, and there were fatal outcomes for 5 patients with TEAE, but none were ascribed to indatuximab ravtansine.

In the second work [43], Jagannath et al. reported the results from 2 clinical trials on 67 RRMM patients. The most common AEs were diarrhea and fatigue. Stable disease was achieved by over 75% of patients, with a 3 month median time to progression and 26.7 months OS. The ADC lorvotuzumab, an anti-CD56 linked to mertansine, was studied by Ailawadhi et al. [44], that on 37 RRMM patients observed a low rate of 3–4 grade AEs, no treatment-related reactions, and no humoral responses against the studied antibody.

### 3.2. Bispecific Antibodies

Bispecific antibodies (BiAbs) bind at the same time to a tumoral antigen and to cytotoxic immune cells, such as T cells and NK cells. Immune effectors are, thus, activated, and able to kill the nearby tumor cells [45]. Even if different bispecific antibodies can be described, the two main classes are those with an Fc region and those without. Bispecific antibodies without an Fc region are small and can easily penetrate tumor tissues [46]. The main negative side is their short half-life, which means that they require frequent administration. This problem can be overcome by using many “half-life extenders”, including polyethylene glycol, albumin-binding moieties, or polyethylene glycol–mimetic polypeptides. Bispecific antibodies with an Fc region are bigger, have a longer half-life, and, as an added advantage, they make possible immune responses Fc-mediated, such as antibody dependent cellular cytotoxicity and complement fixation [47]. The first bispecific antibody for which data in MM treatment were reported is AMG420, an antibody that targets BCMAxCD3. In a phase 1 study, Topp et al. [48] assessed 42 RRMM patients, reporting 2 non-treatment-related deaths from influenza/aspergillosis and adenovirus-related hepatitis. Serious AEs included 20 infections and 14 polyneuropathies, while no central nervous system toxicities or anti-AMG420 antibodies were detected. The OR was 31%, and at the MTD of 400 mg/d there was a 70% response rate. Two other antibodies studied on MM patients are JNJ-64407564 talquetamab and JNJ-64007957 teclistamab. Chari et al. [49], in a phase 1 study, evaluated the use of talquetamab intravenously or subcutaneously administered in 232 RRMM. Their results showed that at the subcutaneous recommended doses of 405 μg/kg/week (30 patients) or 800 μg/kg/every other week (44 patients), common AEs were cytokine release syndrome (CRS) (77% and 80%, respectively), skin-related events (63% and 57%, respectively), and dysgeusia (63% and 57%, respectively). One patient treated every other week developed a dose-limiting toxic effect of grade 3 rash. Moreover, the percentage of responder patients was 70% at the 11.7 month follow-up for patients who were treated weekly and 64% at the 4.2 month follow-up for patients treated every other week. At the 11.7 month follow-up, the median response durations were 10.2 and 7.8 months, respectively. Teclistamab was assessed in two studies by Moreau et al. [50] and Usami et al. [51]. In the first one [50], 165 RRMM patients treated with JNJ-64007957 teclistamab were evaluated, reporting 63% OR at 14.1 months, with 65 patients showing a complete response or better. Patients showed CRS, neutropenia, anemia, and thrombocytopenia as common AEs, and had a median duration of response of 18.4 months and a median PFS of 11.3 months. Moreover, 76.4% of patients displayed infections and 14.5% displayed neurotoxic events, including the effector cell-associated neurotoxicity syndrome (ICANS) shown by five patients. In the second paper [51], 157 patients were treated with at least one dose of teclistamab, and 40 patients were administered the recommended phase 2 dose (subcutaneous 1500 µg/kg/once a week after step-up doses of 60 µg/kg and 300 µg/kg). No dose-limiting toxicities at the recommended phase 2 dose in part 1 were observed. The 40 patients treated with the recommended phase 2 dose showed CRS (28 patients) and neutropenia (26 patients). The OR was 65%; moreover, the median duration of response was not reached using the phase 2 recommended dose, and although exposure to teclistamab was maintained above target exposure levels, the median duration of response was not reached. At 7.1 months median follow-up, the patients alive and continuing treatment were 22 over 26.

### 3.3. Trispecific Antibodies

The development of trispecific antibodies is still in the preclinical stage (Table 1). As mentioned above, bispecific antibodies normally target a tumor antigen and CD3, in order to activate a cytotoxic T cells response against cancer cells. However, without co-stimulation, anergy with a suboptimal reaction against the tumor is very likely [52]. Recently, Wu et al. [53] showed that a trispecific antibody (Figure 2) directed against CD38, CD3, and CD28, which is a recognized T cells co-stimulatory protein, had a killing capacity in CD38+ myeloma cell lines 3–4 log higher than daratumumab. Therefore, MM growth in mice was repressed by the trispecific molecule, while in primates trispecific antibodies promoted memory and effector T cells proliferation, and downregulation of regulatory T cells. Moreover, researchers are working to produce trispecific antibodies able to engage NK cells targeting CD16A, BCMA, and CD200. As a consequence, clinical trials of trispecific antibodies represent an intriguing prospect for the treatment of MM and are eagerly awaited.

## 4. Chimeric Antigen Receptor (CAR) T-Cells

CAR-T cells are one of the most encouraging immunological strategies for the therapies of RRMM patients. They were proven to prolong survival in patients with hematologic malignancies, even after other standard therapeutic methods had failed [54]. CARs are artificial proteins made of a transmembrane domain, an intracellular signaling motif, and an extracellular tumor-specific antibody [55]. This last region is the main point in antigen targeting and is formed by a single-chain fragment (scFv) that derives from natural tumor-specific antibodies [56]. This component is responsible for the binding of CAR-T cells to cancer cells (Figure 2), with consequent T cell activation, proliferation, production of cytokines, and cytolytic degranulation [57]. CAR-T cells are obtained by drawing a peripheral blood sample from the patient; T cells are isolated and then genetically modified to present CARs that are able to recognize a specific TAA. After their expansion, the CAR-T cells are infused into the patient. The TAAs’ recognition induces the activation of signaling pathways in T cells that lead to the production of several pro-inflammatory cytokines (IFN-γ, TNF-α, IL-6, and IL-2) and to the cytolysis of cancer cells [58]. Excellent targets for CAR-T cells therapy are BCMA and CD38, a cell surface protein highly represented in MM cells [59].

Several studies focused their attention on the anti-BCMA CAR-T cells in RRMM patients (Table 2 and Table 3). In a phase 1 study on 25 patients treated with BCMA-targeted CAR-T, Cohen et al. [60] reported reversible grade 3–4 CRS and reversible neurotoxicity in eight patients. Moreover, one patient died at day 24 as a result of severe CRS and encephalopathy. Response treatment from partial to complete was observed in 12 patients, and responder patients showed a decrease in BCMA expression on residual MM cells. Du et al. [61] reported on 49 RRMM patients, with CRS in 17 patients, an OR of 77% with a complete response in 47% of patients. Moreover, median OS and PFS were 29 months and 10 months respectively. Another phase 1 study on 13 RRMM patients [62] showed only 1 case of CRS with grade >3 and ICANS, while there were no atypical neurological toxicities and Parkinson-like movement disorders. Garfall et al. [63] conducted a phase 1 clinical trial on MM patients treated with anti-BCMA CAR-T with or without anti-CD19 CAR-T. A high-grade CRS and only one episode of low-grade neurologic toxicity was observed. Moreover, there was no significant difference between therapy with anti-BCMA + anti-CD19 or with CAR-T-BCMA alone. Li et al. [64] studied the hematologic toxicity (HT) on 54 patients with RRMM treated with a combined infusion of anti-CD19 and anti-BCMA CAR-T cells. Results showed severe neutropenia in 28 patients, severe anemia in 15 patients, and severe thrombocytopenia in 18 patients. They also reported that 28 patients showed prolonged HT (PHT) at 28 days post-infusion and a lower median PFS and OS compared with patients without PHT, suggesting that an early identification and management of PHT would help to prevent life-threatening complications after CAR-T cell therapy, thus, improving patients’ survival. Tang et al. [65] observed that 13 RRMM patients over 16 had stringent complete response when treated with anti-BCMA/CD19 CAR-T; partial response was found in 1 patient and no response was registered in 2 patients. The 11.5 month follow-up showed no relapse in 10 patients, while 4 were dead.

One-year OS and PFS rates were 75% and 68.8% respectively. Moreover, 12 patients had CRS of various grades, and all 16 patients had cytopenia. The combination of anti-CD19 and anti-BCMA was also evaluated in two more papers. Wang et al. [66], in 54 RRMM, observed CRS in 100% of patients, with grade 1–2 in 47 patients and grade 3–5 in 5 patients. The mild CRS group showed 18.2 months PFS, while OS was not reached yet. In the severe CRS, PFS and OS median rates were 1.9 months. Moreover, there was no association between bone marrow tumors and CRS, while a correlation was shown between the grade of CRS and the levels of six serum cytokines, including IL-6, IL-8, IP-10, MIP-1a, and RANTES. Yan et al. [67] studied the response to anti-CD19 and anti-BCMA CAR-T therapy on 21 patients. A stringent complete response was observed in nine patients, complete response in three, a very good partial response in five, and three patients showed a partial response. CRS was observed in 19 patients, and 20 patients showed hematological toxicities, including neutropenia, anemia, and thrombocytopenia. One patient died from a cerebral hemorrhage related to sustained thrombocytopenia, and no deaths were judged as treatment-related. Two more studies evaluated the combination therapy of the CAR-T cells anti-CD38 and anti-BCMA. In the first one, Mei et al. [68] reported that 20 out of 23 patients developed CRS (mostly grade 1–2), 96% developed HT, while neurotoxicity did not occur. Moreover, they observed 17.2 month median PFS. BCMA and CD38 expression on MM cells was maintained in two relapsed patients. Lastly, BM38 CAR-Ts cells were present in 77.8% of evaluable patients at 9 months and 62.2% at 12 months. In the second study, Zhang et al. [69] observed a 90.9% ORR, with a stringent complete response/complete response (sCR/CR) reached by 12 patients. OS and PFS rates at 24 months were 56.6% and 48.7%, respectively. They also observed that 16 patients had a grade 1–2 CRS and 6 patients a grade 3, while 3 patients developed grade 1–2 ICANs.

## 5. Discussion and Conclusions

MM represent 1% of all cancers and is the second most common hematological malignant disease, with an incidence of 4.5–6/100.000/year. Until the early 2000s, patient survival rate at 5 years was <5.6%. During the last 20 years, there have been improvements in clinical outcomes, OS, and PFS, but MM is still hard to treat, with a remitting and relapsing evolution which needs continuous therapy [70]. In this review, we analyzed the most recent work on emerging new treatments for MM, such as neoantigen vaccination strategies, ADCs, bispecific and trispecific antibodies, and CARs-T cells.

Neoantigens are abnormally-expressed peptides that originate from the genetic instability always present during carcinogenesis. They have attracted the attention of experts in immunotherapy because they are recognized as non-self by the immune system, which explains the lack of tolerance and autoimmunity reactions caused by neoantigen-specific immune reactions. Recently, Xie et al. [71], on the basis of previous studies [22], reported the presence of two potential neoantigens, UBR4 and PRKDC, in patients with multiple myeloma. However, some limiting factors in the development and use of neoantigens exists, such as (i) the scarce amount of antigens found in tumors actually meeting the neoantigen criteria; (ii) a lack of efficient neoantigen screening methods; (iii) the large amount of time required by a cycle of neoantigen vaccines, which makes them impossible to use in clinical trials for patients with a short survival period; (iv) difficulties in developing vaccine preparation and delivery methods, particularly for therapies using small nucleic acid therapies [72].

ADCs and bispecific and trispecific antibodies are other promising therapy strategies that aim to potentiate the tumor immunological response by adding a cytotoxic agent to the antibody in ADCs, or simultaneously binding a tumoral antigen and an immune cell with cytotoxic effects, with the addition of a co-stimulatory factor (trispecific antibodies) or without one (bispecific antibodies). While trispecific antibodies are still in the preclinical stage of development, the examined studies on ADCs and bispecific antibodies showed an overall good tolerance in patients, with ORRs always >50%, and neutropenia, anemia, thrombocytopenia, polyneuropathy, corneal toxicity, and CRS as the most common AEs. Despite these encouraging results, more research is needed to determine the best way to sequence or combine these molecules with the currently available therapies [70].

CAR-T cells are T cells engineered with chimeric antigen receptors, artificial proteins that permit CAR-T cells to bind cancer cells, causing T cells activation, proliferation, production of cytokines, and cytolytic degranulation. Data from clinical trials on MM patients treated with CAR-T cells showed CRS as the most common AE, with different grades of gravity in different studies. Other common AEs were neurotoxicity, immune ICANS, neurological toxicities, infections, Parkinson-like movement disorders, and HTs, such as anemia and a reduction in neutrophils and platelets. Despite the demonstrated responses in RRMM patients, CAR-T cells therapies have some challenges, such as (i) their availability and the prolonged manufacturing times; (ii) the use of CAR-T needs to be associated with the therapy for lymphodepletion; (iii) highly frequent inflammatory, hematologic, and neurologic toxicities; (iv) a better understanding of resistance mechanisms, the definition of the proper approaches after progression and the best arrangement of other anti-BCMA therapies [73].

However, single treatments nearly always showed worse results than combinations, as MM is a genetically and immunologically heterogeneous disease. Identifying the best immunotherapy companion medicines should lead to a better understanding of the resistance or non-response mechanisms in patients treated with monotherapy. There are a lot of options to think about. Studies combining gamma secretase inhibitor (GSI) with CAR-T, BiAbs, and ADC are currently being conducted. GSI may aid in increasing the expression of BCMA on tumor cells and reducing the amounts of soluble antigens in circulation. Moreover, PD-1, LAG-3, TIM-3, and TIGIT are only a few immune checkpoints being researched in MM; however, it is not known how these immune interventions affect disease progression [74,75,76]. Furthermore, as was previously mentioned, increased levels of Tregs can make BiAbs less effective. CD38^+^ myeloid-derived suppressor cells, B regs, and Tregs have been depleted in vitro by daratumumab, while T cell clonal expansion was promoted by this antibody. A combination of daratumumab with teclistamab and talquetamab is currently being studied [77,78]. Combination therapy, however, might potentially result in unique or unexpected toxicities; therefore, this must be kept in mind. Lastly, bioenergetic processes in tumor cells has been disrupted by immunoediting trains, being able to neutralize T cell-mediated immunosurveillance by compelling a metabolic tug-of-war between invading T cells and the tumor and creating a microenvironment with suppressive action on the tumor.

The reprogramming of cellular metabolism could be the next frontier. According to recent information, metabolic reprogramming may affect the activation, differentiation, function, and exhaustion of T lymphocytes. Proper activation of dormant T cells encourages the transition of effector T cells from catabolic and oxidative metabolism to aerobic glycolysis and then back to oxidative metabolism. These metabolic changes have a significant impact on how T cell differentiation and fate are determined. T cells metabolic processes, however, may be dysregulated as a result of interactions with tumors or with the tumor microenvironment (TME). Significantly, a novel mechanism that causes a significant inhibition of effector T cells is the metabolic competition in the tumor environment. It is understood that metabolic reprogramming can be targeted as a promising strategy to disrupt tumor cells’ hypermetabolic condition and improve the immune system’s ability to take in nutrients. Immunotherapies, such as immune checkpoint inhibitors, adoptive cell therapy, and oncolytic virus therapy, can also considerably influence solid tumors therapy. However, not all patients showed an adequate response to the above-mentioned therapies. To better regulate T cell anti-tumor response, it seems necessary to understand how immunotherapy interferes with T cell metabolism [79].

In conclusion, although emerging new strategies have improved OS and PFS rates in MM patients, much work is still needed to improve control and relapse/resistance rates of a not yet curable disease.

## Figures and Tables

**Table 1:** Summary of the studies on neoantigens in MM patients.

Study	Number of Patients	Results	Reference Number
Miller et al. 2017	664	Shorter PFS in patients with a higher neoantigen load	[33]
Dong et al. 2021	893	Worse OS in patients with high levels of neoantigens	[34]
Perumal et al. 2020	184	Higher neoantigen burden in relapsed patients	[22]
Jian et al. 2022	478	High NAIRscore associated with increased OR	[35]

**Table 2:** Summary of studies on drug-conjugated, bispecific and trispecific antibodies in MM patients.

Study	Number of Patients	Drug	Type of Antibody	Target	Results	Reference Number
Trudel et al. 2018	73	Belantamab mafodotin (GSK2857916)	ADC	BCMA	AEs: corneal events, thrombocytopenia, and anemia; 12 serious AEs treatment-related; no treatment-related deaths; OR 60%.	[40]
Trudel et al. 2019	35	Belantamab mafodotin (GSK2857916)	ADC	BCMA	OR: 60% median PFS: 12 months; median duration of response: 14.3 months; AEs: platelets reduction and corneal damages	[41]
Kelly et al. 2021	64	Indatuximab ravtansine	ADC	CD138	MTD of indatuximab ravtansine plus lenalidomide: 100 mg/m^2^; OR in the indatuximab ravtansine + lenalidomide group: 71.7%; OR in indatuximab ravtansine + pomalidomide group: 70.6%; positive responses in indatuximab ravtansine + lenalidomide group: 85%; clinical benefit response in indatuximab ravtansine plus pomalidomide group: 88%; AEs: anemia, platelets, and neutrophils reduction.	[42]
Jagannath et al. 2019	67	Indatuximab ravtansine	ADC	CD138	AEs: diarrhea and fatigue; stable disease achieved by over 75% of patients; OS: 26.7 months.	[43]
Ailawadhi et al. 2019	37	lorvotuzumab mertansine	ADC	CD56	Few grade 3–4 AEs; no infusion-related reactions; the studied drug induced no humoral responses.	[44]
Topp et al. 2020	42	AMG420	Bispecific	BCMAx CD3	Two nontreatment-related deaths due to influenza/aspergillosis and adenovirus-related hepatitis; AEs: infections, polyneuropathy; no CNS toxicities or anti-AMG 420 antibodies; OR: 31%; 70% response rate at the maximum tolerated dose (MTD) of 400 mg/d.	[48]
Chari et al. 2022	232	Talquetamab	Bispecific	GPRC5dxCD3	AEs: CRS, skin-related, dysgeusia One patient developed a dose-limiting toxic effect of grade 3 rash; OR at 11.7 follow-up: 70% at 11.7 follow-up for patients who were treated weekly; OR at 4.2 months follow-up: 64% for patients treated every other week.	[49]
Moreau et al. 2022	165	Teclistamab	Bispecific	BCMAx CD3	OR at 14.1 months: 63%; median duration of response: 18.4 months; median PFS: 11.3 months; AEs: CRS, neutropenia, anemia, thrombocytopenia, infections, neurotoxic events.	[50]
Usmani et al. 2021	157	Teclistamab	Bispecific	BCMAx CD3	No dose-limiting toxicities at the recommended phase 2 dose in part 1; AEs: CRS, neutrophils reduction; OR: 65%; not reached median duration of response at the recommended phase 2 dose.	[51]

**Table 3:** Summary of the studies on CAR-T cells in MM patients.

Study	Number of Patients	Target	Results	Reference Number
Cohen et al. 2019	25	BCMA	AEs: CRS, reversible neurotoxicity; 1 patient died at day 24 from severe CRS and encephalopathy.	[60]
Du et al. 2022	49	BCMA	AEs: CRS, OR: 77%; median OS: 29 months; median PFS:10 months.	[61]
Frigault et al. 2023	13	BCMA/CD19	AEs: 1 case of CRS with grade > 3, ICANS.	[62]
Garfall et al. 2022	30	BCMA/CD19	AEs: CRS, 1 patient with low-grade neurologic toxicity; no significant difference between therapy with anti-BCMA + anti-CD19 or with CAR-T-BCMA alone.	[63]
Li et al. 2022	54	BCMA/CD19	AEs: neutropenia, anemia, thrombocytopenia; 28 patients had PHT 28 days post-infusion and showed a lower median PFS and OS than patients without PHT.	[64]
Tang et al. 2022	16	BCMA/CD19 CAR-T	Complete response in 13 patients, partial response in 1 patient, no response in 2 patients; one year OS and PFS: 75% and 68.8; AEs: CRS of various grades, cytopenia.	[65]
Wang et al. 2022	54	BCMA/CD19 CAR-T	100% incidence of CRS; PFS in the mild CRS group: 18.2 months; OS in mild CRS group: not reached yet; PFS and OS in the severe CRS group: 1.9 months; no association between bone marrow tumors and CRS.	[66]
Yan et al. 2019	21	Anti-BCMA, anti-CD19 and anti-CD20 CAR-T	Stringent complete responses in nine patients, complete responses in three patients, very good partial response in five patients, partial responses in three patients; AEs: CRS, neutropenia, anemia, thrombocytopenia; one patient died from a cerebral hemorrhage.	[67]
Mei et al. 2021	23	Anti-BCMA/CD38 CAR-T	AEs: CRS, HT; median PFS: 17.2 months; BCMA and CD38 expression observed in two relapsed patients on MM cells; BM38 CAR-Ts cells visible in 77.8% of evaluable patients at 9 months and 62.2% at 12 months.	[68]
Zhang et al. 2022	22	BCMA/CD38	OR: 90.9%; OS and PFS at 24 months: 56.6% and 48.7%; AEs: CRS, ICANs.	[69]
